# Interimplant Papilla Reconstruction by Using Demineralized Freeze Dried Bone Allograft Block Fixed by Titanium Screw: A Case Report

**DOI:** 10.1155/2012/809347

**Published:** 2012-12-10

**Authors:** Preeti Charde, M. L. Bhongade, Aniruddha Deshpande, Anendd Jadhav, Kaustubh Thakare, Priyanka Jaiswal

**Affiliations:** ^1^Department of Periodontology and Implantology, Sharad Pawar Dental College and Hospital, Wardha 442101, India; ^2^Department of Periodontology and Implantology, CSMSS Dental College and Hospital, Aurangabad 442001, India; ^3^Department of Oral and Maxillofacial Surgery, Sharad Pawar Dental College and Hospital, Wardha 442101, India

## Abstract

Dental implants are now considered as a predictable treatment modality for the oral rehabilitation of partially or fully edentulous patients. Recently emphasis has changed towards achieving a predictable esthetic success. Creating aesthetically successful implant-supported restoration in the anterior region of oral cavity depends on the presence of interimplant papilla when multiple implants are used. The present paper reports a case of interimplant papilla reconstruction in esthetic zone of maxilla during one stage early loading multiple implant procedure using demineralized freeze dried bone allograft block fixed by titanium screw.

## 1. Introduction

Dental implants are now considered as a predictable treatment modality for the oral rehabilitation of partially or fully edentulous patients. Initially, the factors considered while evaluating success included direct contact between alveolar-supporting bone and dental implants, together with a lack of clinical and radiographic signs of inflammation but, with the growing use of implant-supported oral rehabilitation in the partially edentulous patient and single tooth restoration, emphasis has changed towards achieving predictable esthetic success particularly in anterior region of oral cavity. In the anterior part of mouth, however, there is often a noticeable difference in the soft tissue appearance between dental implants. Deficiency of soft tissue between implant-supported restorations often affects the final aesthetic result. This is particularly important in those patients who may show peri-implant soft tissue when smiling or speaking [1]. Creating aesthetically successful implant-supported restoration in the anterior region of oral cavity depends on the presence of interimplant papilla when multiple implants are used. 

The absence of the interimplant papilla can lead to cosmetic deformities, phonetic difficulty, and food impaction. However, reconstructing a predictable peri-implant papilla is the most complex and challenging aspect of implant dentistry in particular, when two or more adjacent implants are placed. In addition, loss of the vertical dimension of the edentulous ridge may further complicate papilla reconstruction. 

 The importance of the presence of bone medially between implants in an architecture that supports the soft tissue and allows it to conform to the desired shape of the interdental papilla is, therefore, the key to successful papilla reconstruction between dental implants. For reconstruction of interimplant papilla most successful and predictable aesthetics results can be accomplished only when underlying labial and interproximal osseous support is therapeutically provided [2].

Given the fact that osseous support is crucial for the soft tissue profile between the implants, the present paper reports a case of interimplant papilla in esthetic zone of maxilla during one stage early loading multiple implant procedure using demineralized freeze dried bone allograft block fixed by titanium screw (see Figure 4).

## 2. Case Presentation

A systemically healthy 21-year-old male patient reported to the Department of Periodontology and Implantology with chief complaint of missing teeth in maxillary anterior region of jaw. Intraoral examination revealed missing 11 and 21. After clinical and radiographic evaluation replacement of missing teeth by one stage early loading implants along with reconstruction interimplant papilla using demineralized freeze dried bone allograft block fixed by titanium screw was planned. Prior to surgical procedure interimplant papilla measurements including measurement of papillary height using Grossberg criteria (2001) [1] and measurement of papilla contour using Jemt index (1997) [3] was carried out. Radiographic examination using intraoral periapical radiograph (IOPA) with long cone (XCP Rinn, Dentsply, New York, USA) paralleling technique was carried out to measure the vertical crestal bone level between implants, which was calculated from contact point after placement of restoration to the highest coronal point of crestal bone between implants [4, 5]. All measurements were recorded at baseline and again postoperatively at 3 months and at 6 months after final restoration. 

### 2.1. Implant Placement Procedure

**Figure 1:**
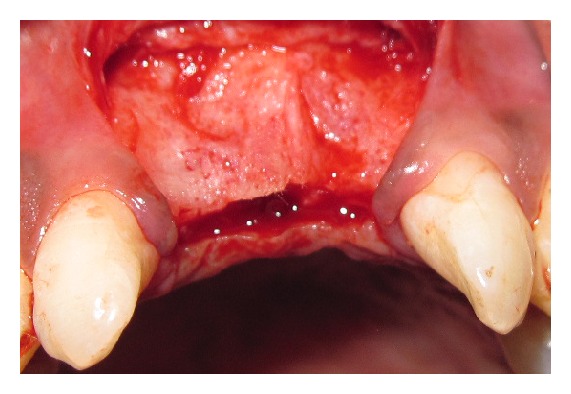
After reflection of flap.

 Briefly after induction of local anesthesia, horizontal palatal incision was made 2 mm away from the crest of the ridge using bard parker surgical blade number 15, without splitting the adjacent papillae, followed by vertical releasing incision on labial surface made extending to the vestibule. The papillae of the adjacent teeth were not included in the flap design (see Figure 1). A full thickness flap was raised labially and palatally exposing the underlying ridge of the implant site. Asurgical drill guide was used for the precise placement of the pilot drill. After pilot drill application, the implants site was prepared with the corresponding size of parallel drill. The implants were placed in the recipient site by means of an insertion device, and a torque driver set at 35 Ncm was used to evaluate primary stability of implant. The implant neck was positioned at the crestal bone level or slightly submerged. The healing abutment extension of the implant was placed in such a way that the head of the implant protrudes about 2 to 3 mm from the bone crest. 

### 2.2. Papilla Reconstruction Procedure

**Figure 2:**
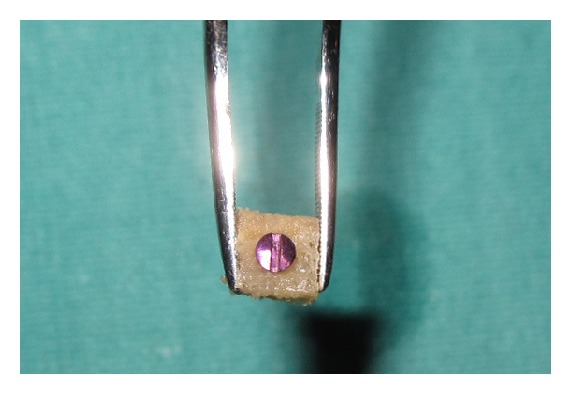
Prepared bone block predrill and adapted Titanium screw.

Both implants were placed in such way that the interimplant distance was ≥3 mm to ensure sufficient blood supply to the interimplant bone after placing papillary titanium screw with alloplastic bone block between the two implants. 1 mm diameter bur was used to drill midway between the two implants. A bone block allograft (freeze, dried, demineralized, irradiated bone allograft block) was hydrated with a sterile saline solution for at least 45 minutes before use. Then it was trimmed with a fissure bur in a high speed hand piece with a copious saline solution to remove residual bone particles. The prepared block allograft was predrilled to accommodate (1.5 mm × 8 mm) titanium screw (see Figure 2). Fixation screws were placed in a prepared block allograft with an oblique fashion so as not to induce stress fracture in the allograft. After stabilization of allograft block between the two implants, the buccal flap was positioned around the implants and sutured to the palatal flap. Complete tension free soft tissue closure was achieved and a provisional restoration was cemented. Patient received antibiotics (Amoxicillin PO 500 mg t.i.d.) and analgesics (Ibuprofen PO 400 mg, t.i.d.) after surgery were continued for at least 5 days postsurgically. Patient was instructed not to brush in the treated area but to rinse 3 times per day for 1 minute with chlorhexidine digluconate of 0.12% until suture removal. Sutures were removed within 7 to 10 days of implantation and, during the same visit, the final impression was registered using high-viscosity vinyl polysiloxane and within 15 days a definitive customized abutment with gingival emergence was established by permanent restoration using a metal-ceramic restoration. The patients were recalled at 3 months and 6 months following permanent restoration. At each recall visit clinical and radiographic measurements were recorded preoperatively and were repeated at 3 months and 6 months after final restoration.

In the present case report at 6-month followup, papillary height was 2.00 mm at baseline, which was increased to 3.8 mm at 3 months after papilla reconstruction procedure with a gain of 1.8 mm. At 6 months, it was further increased to 4 mm with gain of 2 mm. At 6-month measurement of papilla contour using Jemt index (1997) [3] was score 3 which indicates complete reconstruction of interimplant papilla. Radiographically the distance between contact point after placement of restoration to the highest coronal point of crestal bone between implants was less than 5 mm.

## 3. Discussion

Today, with the high survival and success rates of implant therapy, the focus has shifted toward creating an aesthetic restoration that is indistinguishable from natural teeth and remains stable over time. Construction of an aesthetically pleasing restoration involves not only harmonizing the size, shape, position, and colour of each prosthetic tooth with the adjacent teeth but also establishing peri-implant soft tissue compatibility with the surrounding gingiva and mucosa (see Figure 5). This is particularly important in esthetic zone of maxilla. 

The use of dental implants in the anterior region of mouth is a technique sensitive procedure. The placement of implants in an ideal position for an anterior restoration is not often possible because of the lack of sufficient bone and soft tissue defects. In addition, management of the papilla during implant placement does not always allow predictable soft tissue wound healing and esthetic integration of the prosthetic crown. This may result in esthetic failures. 

**Figure 3:**
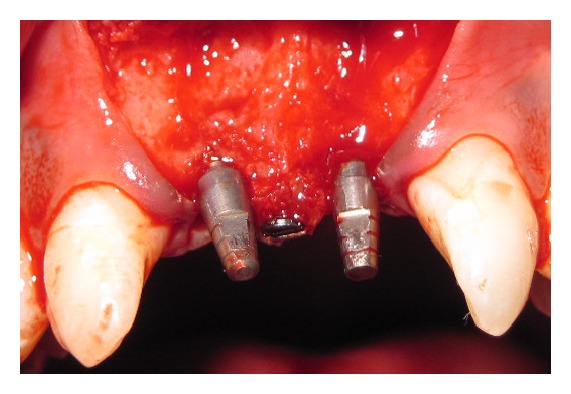
Between implants bone block fixed by using Titanium screw.

**Figure 4:**
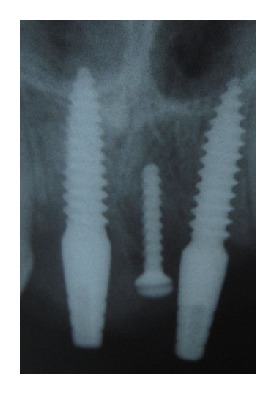
postoperative radiograph showing implant and screw.

**Figure 5:**
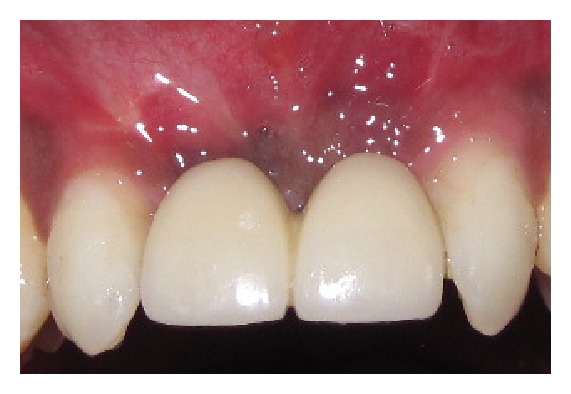
Implant with final prosthesis at 6 months.

El Askary (2000) [6] in a case report study used interimplant papillary template which carries the bone grafting mixture for reconstruction of interimplant papilla. They reported significant amount of bone regeneration between implants to support newly generated interimplant papilla. El Askary (2000) [7] in a case report used titanium papillary insert composed of a pyramidal shaped polished titanium core, which has a height of 2 to 3 mm and base of 3 mm buccolingually and 1 mm in mesiodistal dimension. They reported complete interimplant papilla reconstruction in 3 patients and suggested that use of titanium papillary insert could help with the establishment of interimplant papilla in those cases, when it was placed simultaneously with dental implants. Therefore, the problems of soft tissue quantity and quality are usually managed before or during implant therapy to enhance the soft tissue-implant-supported restoration interface. In the present case report, papilla reconstruction was performed using DFDBA block fixed by titanium screw during implant placement (see Figure 3).

## 4. Conclusion

In present case report complete reconstruction of interimplant papilla suggested that the use of demineralized freeze dried bone allograft block fixed by titanium screw is found to be effective in the reconstruction of interimplant papilla in those cases, when it was placed simultaneously with dental implants.
